# Evening Primrose (*Oenothera biennis*) Biological Activity Dependent on Chemical Composition

**DOI:** 10.3390/antiox7080108

**Published:** 2018-08-14

**Authors:** Magdalena Timoszuk, Katarzyna Bielawska, Elżbieta Skrzydlewska

**Affiliations:** Department of Inorganic and Analytical Chemistry, Medical University of Bialystok, 15-089 Bialystok, Poland; magdalena.timoszuk@umb.edu.pl (M.T.); katarzyna.bielawska@umb.edu.pl (K.B.)

**Keywords:** evening primrose oil, γ-linolenic acid, linoleic acid, omega-6 fatty acids, eicosanoids

## Abstract

Evening primrose (*Oenothera* L.) is a plant belonging to the family Onagraceae, in which the most numerous species is *Oenothera biennis*. Some plants belonging to the genus *Oenothera* L. are characterized by biological activity. Therefore, studies were conducted to determine the dependence of biological activity on the chemical composition of various parts of the evening primrose, mainly leaves, stems, and seeds. Common components of all parts of the *Oenothera biennis* plants are fatty acids, phenolic acids, and flavonoids. In contrast, primrose seeds also contain proteins, carbohydrates, minerals, and vitamins. Therefore, it is believed that the most interesting sources of biologically active compounds are the seeds and, above all, evening primrose seed oil. This oil contains mainly aliphatic alcohols, fatty acids, sterols, and polyphenols. Evening primrose oil (EPO) is extremely high in linoleic acid (LA) (70–74%) and γ-linolenic acid (GLA) (8–10%), which may contribute to the proper functioning of human tissues because they are precursors of anti-inflammatory eicosanoids. EPO supplementation results in an increase in plasma levels of γ-linolenic acid and its metabolite dihomo-γ-linolenic acid (DGLA). This compound is oxidized by lipoxygenase (15-LOX) to 15-hydroxyeicosatrienoic acid (15-HETrE) or, under the influence of cyclooxygenase (COX), DGLA is metabolized to series 1 prostaglandins. These compounds have anti-inflammatory and anti-proliferative properties. Furthermore, 15-HETrE blocks the conversion of arachidonic acid (AA) to leukotriene A_4_ (LTA_4_) by direct inhibition of 5-LOX. In addition, γ-linolenic acid suppresses inflammation mediators such as interleukin 1β (IL-1β), interleukin 6 (IL-6), and cytokine - tumor necrosis factor α (TNF-α). The beneficial effects of EPO have been demonstrated in the case of atopic dermatitis, psoriasis, Sjögren’s syndrome, asthma, and anti-cancer therapy.

## 1. Introduction

Evening primrose (*Oenothera* L.) is a plant belonging to the Onagraceae family. There are about 145 species in the genus *Oenothera* L., occurring in the temperate and tropical climate zones of North and South America. Some species have adapted to new areas, inhabiting the countries of the European continent, and about 70 species are now present in Europe. The most numerous species in the *Oenothera* L. family is *Oenothera biennis*, which also has the best-studied biological activity. It has been indicated that *Oenothera biennis* is beneficial in the treatment of many diseases. Therefore, research is ongoing to determine the chemical composition of these plants and how it relates to the biological activity of evening primrose. This research mainly concerns extracts from various parts of evening primrose (e.g., the leaves, stems, and seeds) [1].

## 2. Chemical Composition of Evening Primrose (*Oenothera biennis*)

Methanolic extracts prepared from the aerial parts of *Oenothera biennis* contain mainly phenolic acids and flavonoids. Phenolic acids present in the analyzed extracts include the following compounds: gallic acid and its ester derivatives (e.g., methyl gallate, galloylglucose, digalloylglucose, and tris-galloylglucose), 3-*p*-feruloylquinic acid, 3-*p*-coumaroylquinic acid, 4-*p*-feruloylquinic acid, caffeic acid pentoside, ellagic acid and its ester derivatives (e.g., ellagic acid hexoside and ellagic acid pentoside), and valoneic acid dilactone. Flavonoids present in the extracts include the following compounds: myricetin 3-*O*-glucuronide, quercetin 3-*O*-galactoside, quercetin 3-*O*-glucuronide, quercetin 3-*O*-glucoside, quercetin pentoside, quercetin dihexoside, quercetin glucuronylhexoside, quercetin 3-*O*-(2’-galloyl)-glucuronide, kaempferol 3-*O*-rhamnoglucoside, kaempferol 3-*O*-glucoside, kaempferol 3-*O*-glucuronide, kaempferol 3-*O*-(2’-galloyl)-glucuronide, and kaempferol pentoside [2].

The aqueous leaf extract of *Oenothera biennis* contains phenolic compounds (e.g., ellagitannins and caffeoyl tartaric acid) and flavonoids (quercetin glucuronide and kaempferol glucuronide) [3]. Among the tannins contained in the leaves of the evening primrose are oenothein A and oenothein B. The carbohydrates present in the extracts include arabinose, galactose, glucose, mannose, galacturonic acid, and glucuronic acid.

The roots of evening primrose contain the following sterols: sitosterol, oenotheralanosterol A, and oenotheralanosterol B. The triterpenes maslinic acid and oleanolic acid are also present in the root, along with the following carbohydrates: arabinose, galactose, glucose, mannose, galacturonic acid, and glucuronic acid. The following tannins are also found: gallic acid, tetramethylellagic acid, oenostacin, and 2,7,8-trimethylellagic acid [4]. The methanolic extract of the *Oenothera biennis* root also possesses significant amounts of xanthone (9H-xanthen-9-one) and its derivatives, such as dihydroxyprenyl xanthone and cetoleilyl diglucoside, which possess diverse biological and pharmacological properties [5].

Evening primrose seeds contain about 20% oil. The amount of oil depends on various factors, such as the age of the seed, the cultivar, and the growth conditions [6].

Generally, evening primrose oil is obtained from *Oenothera biennis* seeds using the cold-pressing method. The oil is a blend of about 13 triacylglycerol fractions, where the dominant combinations consist of the following fatty acids: linoleic–linoleic–linoleic (LLL, 40%), linoleic–linoleic–γ-linolenic (LLLnγ, approximately 15%), linoleic–linoleic–palmitic (LLP, approximately 8%), and linoleic–linoleic–oleic (LLO, approximately 8%) [7]. The oil consists of triacylglycerols—about 98%, with a small amount of other lipids and about 1–2% non-saponifiable fraction [6]. 

Evening primrose oil is very high in linoleic (70–74%) and γ-linolenic (8–10%) acids, and also contains other fatty acids: palmitic acid, oleic acid, stearic acid, and (in smaller amounts) myristic acid, oleopalmitic acid, vaccenic acid, eicosanoic acid, and eicosenoic acid (see Table 1) [8]. The phospholipid fraction comprises only 0.05% of the oil, and the following phospholipids have been identified in it: phosphatidylcholines (31.9%), phosphatidylinositols (27.1%), phosphatidylethanolamines (17.6%), phosphatidylglycerols (16.7%), and phosphatidic acids (6.7%) [6].

Evening primrose oil includes aliphatic alcohols, which make up about 798 mg/kg of the oil, 1-tetracosanol (about 237 mg/kg oil), and 1-hexacosanol (about 290 mg/kg oil) being present in the largest amount. The main triterpenes present are β-amyrin (about 996 mg/kg oil) and squalene (about 0.40 mg/kg oil) [8]. The oil contains a small amount of tocopherols: α-tocopherol (76 mg/kg oil), γ-tocopherol (187 mg/kg oil), and δ-tocopherol (15 mg/kg oil) [6].

Evening primrose seeds also contain phenolic acids, which are present in free acid form and as ester and glycoside derivatives (see Table 2) [9]. It has been shown that the seeds contain about 15% protein and 43% carbohydrates (in the form of cellulose, along with starch and dextrin). Lignin is also found in the seeds. In addition, the seeds contain amino acids: tryptophan (1.60%), lysine (0.31%), threonine (0.35%), cysteine (1.68%), valine (0.52%), isoleucine (0.41%), leucine (0.87%), and tyrosine (1.05%). Moreover, the seeds contain minerals, mainly calcium, potassium, and magnesium, and vitamins A, B, C, and E [10]. 

Evening primrose oil also contains polyphenols, such as hydroxytyrosol (1.11 mg/kg oil), vanillic acid (3.27 mg/kg oil), vanillin (17.37 mg/kg oil), *p*-coumaric acid (1.75 mg/kg oil), and ferulic acid (25.23 mg/kg oil) [8].

The unsaponifiable matter of oil is composed partially of sterols, which comprise 53.16% of this fraction (see Table 3) [8].

The seed ash contains a group of macroelements and microelements, including calcium, magnesium, potassium, phosphorus, manganese, iron, sodium, zinc, and copper (see Table 4) [10].

## 3. Biological Activity of Evening Primrose Oil (*Oenothera biennis*)

The biological effect of evening primrose oil is a result of its composition and the biological properties of its components. Since the most important components in terms of quantity are polyunsaturated fatty acids (PUFAs), mainly linoleic acid (LA) and γ-linolenic acid (GLA) which belong to the group of omega-6 acids. The biological significance, especially of these acids, will be discussed in more detail. 

Linoleic acid belongs to the group of essential fatty acids. These are also called exogenous fatty acids, because the human body does not synthesize them and it is necessary to obtain them from food [11]. Evening primrose oil contains over 70% linoleic acid (LA) and about 9% γ-linolenic acid (GLA) [10]. Linoleic acid and γ-linolenic acid contribute to the proper functioning of many tissues of the human body, because they are precursors of compounds that lead to the generation of anti-inflammatory eicosanoids, such as the series 1 prostaglandins and 15-hydroxyeicosatrienoic acid (15-HETrE). On the other hand, the enzymatic conversion of linoleic acid to arachidonic acid (AA) may form pro-inflammatory compounds, such as series 2 prostaglandins and series 4 leukotrienes [11]. With reference to the above, it is suggested that evening primrose oil may influence inflammatory diseases, including skin problems.

Linoleic acid [12], among others, plays an important role in the proper functioning of the skin, especially the stratum corneum, in which it is one of the main components of the ceramides building the lipid layer. It has been shown that the presence of this acid prevents the skin from peeling and the loss of water through the epidermis, while at the same time improving skin softness and elasticity and regulating the process of epidermal keratinization. A deficiency of linoleic acid, which is contained in large quantities in ceramide 1, leads to its replacement by oleic acid. This causes a deterioration in the protective properties of the epidermis [12,13].

Under the influence of Δ-6-desaturase (D6D), linoleic acid undergoes dehydrogenation to form γ-linolenic acid. The activity of Δ-6-desaturase in human cells depends on various factors and is reduced under the influence of nicotine, alcohol, magnesium deficiency, and a poor diet rich in saturated fatty acids, and under conditions of physiological aging of the body [12,14]. The basic component of evening primrose oil is linoleic acid, and the possibility of its metabolism to γ-linolenic acid due to the action of Δ-6-desaturase is an important point. Δ-6-Desaturase (D6D) activity is highest in the liver, the brain, the heart, and lung cells [15]. D6D activity is several times higher in the fetal human liver than in the adult human liver [11]. The fatty acid desaturase 2 (FADS2) gene encoding the Δ-6-desaturase enzyme is also expressed in skin cells: within the sebaceous gland, D6D desaturates palmitic acid to sapienic acid, which is the major fatty acid of human sebum [16,17]. On the other hand, the main epidermal cells—keratinocytes—are characterized by their lack of D6D and D5D activity [18] and the dermal fibroblasts express the D6D mRNA, which is capable of desaturation of the essential fatty acid (EFA) in the skin [19].

Δ-6-Desaturase is also present in evening primrose seeds. Huang et al. (2010) report that the cDNA sequence of the D6D gene was obtained from the developing seeds. The transformation of the plasmid DNA of a *Saccharomyces cerevisiae* strain then showed that after the addition of a medium containing linoleic acid to the yeast cells, a signal was obtained from γ-linolenic acid, indicating the presence of Δ-6-desaturase in seeds of the species *Oenothera biennis* [20,21].

A deficiency of γ-linolenic acid and other metabolites of linoleic acid was demonstrated in the plasma of patients with atopic dermatitis. This is linked to a decrease in Δ-6-desaturase activity, which makes the conversion of linoleic acid to γ-linolenic acid and the formation of its metabolites impossible [12,22]. It was found that oral treatment with evening primrose oil, which contains γ-linolenic acid, may lead to a reduction in the symptoms of atopic dermatitis [22]. However, subsequent studies have not confirmed that oral supplementation with evening primrose oil improves the skin condition in patients with atopic dermatitis [23].

**Figure 1:**
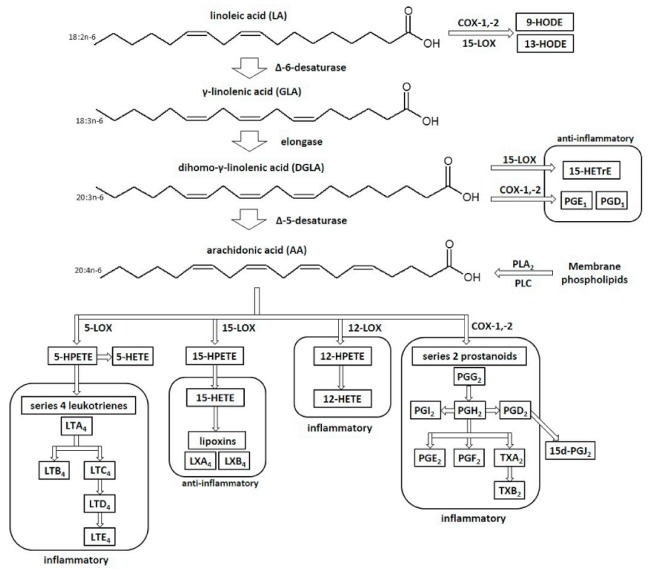
Linoleic acid (LA) metabolism. 9-HODE: 9-hydroxyoctadecadienoic acid; 13-HODE: 13-hydroxyoctadecadienoic acid; 15-HETrE: 15-hydroxyeicosatrienoic; PGE_1_: prostaglandin E1; PGD_1_: prostaglandin D1; PLA_2_: phospholipase A2; PLC: phospholipase C; 5-HPETE: 5-hydroperoxyeicosatetraenoic acid; 5-HETE: 5-hydroxyeicosatetraenoic acid; LTA_4_: leukotriene A4; LTB_4_: leukotriene B4; LTC_4_: leukotriene C4; LTD_4_: leukotriene D4; LTE_4_: leukotriene E4; 15-HPETE: 15-hydroperoxyeicosatetraenoic acid; 15-HETE: 15-hydroxyeicosatetraenoic acid; LXA4: lipoxin A_4_; LXB_4_: lipoxin B4; 12-HPETE: 12-hydroperoxyeicosatetraenoic acid; 12-HETE: 12- hydroxyeicosatetraenoic acid: PGG_2_: prostaglandin G2; PGH_2_: prostaglandin H2; PGI_2_: prostaglandin I_2_: PGD2: prostaglandin D2; 15-d-PGJ_2_: 15-deoxy-delta-12,14-prostaglandin J2; PGE_2_: prostaglandin E2: PGF_2_: prostaglandin F2: TXA_2_: thromboxane A2; TXB_2_: thromboxane B2.

The hydrocarbon chain of the emerging or supplied γ-linolenic acid under the influence of an elongase is elongated to dihomo-γ-linolenic acid (DGLA). The GLA elongation is faster than the desaturation of linoleic acid [24,25]. Dihomo-γ-linolenic acid is metabolized by cyclooxygenase (COX) to series 1 prostaglandins, which are eicosanoids with anti-inflammatory activity (see Table 5) [24]. Under the influence of 15-lipoxygenase (15-LOX), DGLA is oxidized to 15-hydroxyeicosatrienoic (15-HETrE) acid, which has anti-inflammatory and anti-proliferative properties [24,26]. Therefore, increased levels of GLA and DGLA, which are metabolized to anti-inflammatory compounds, suppress the inflammatory reaction. However, a decrease in these acids’ levels may lead to the development of inflammatory diseases [24,27]. In addition, GLA suppresses inflammation mediators such as IL-1β, IL-6, and TNF-α cytokines [12,28]. In contrast, 15-HETrE acid, which is a product of the oxidation of dihomo-γ-linolenic acid by 15-LOX, has the ability to inhibit the synthesis of series 4 leukotrienes, whose elevated levels cause intensified pathological cell hyperproliferation [24]. This contributes to the inhibition of the pro-inflammatory action of leukotrienes, which are involved, among others, in the development of asthma [24,29] (see Figure 1).

Regardless of the metabolism of DGLA catalyzed by COXs and 15-LOX, dihomo-γ-linolenic acid under the influence of Δ-5-desaturase (D5D) is converted to arachidonic acid (AA), which is the precursor of many lipid mediators in the body, mainly pro-inflammatory [18,24]. Under physiological conditions, the sources of AA are membrane phospholipids, from which it is released by the hydrolysis of ester bonds, mainly via the action of phospholipase A_2_ (PLA_2_). There are two main ways that lead to the formation of free arachidonic acid. One pathway leads to the hydrolytic release of AA by the cytosolic isoform of PLA_2_. The second way leads to the release of AA by the indirect action of phospholipase C (PLC) and diacylglycerol (DAG) lipase. DAG lipase and PLC result in the formation of inositol 1,4,5-triphosphate and DAG. The latter is then hydrolyzed by DAG lipase to form free arachidonic acid and monoacylglycerol (MAG) [18,30].

In pathological conditions and, for example, in the case of excessive exposure of the skin to UV radiation, the redox balance is disturbed and oxidative stress occurs, which results in the activation of cytosolic phospholipase A2 (cPLA_2_) and PLC in the skin cells [18]. This leads to the excessive release of AA and the increased production of eicosanoids via cyclooxygenases and lipoxygenases. COX-1 and COX-2 catalyze the transformation of arachidonic acid into the series 2 prostanoids (PGE_2_, PGD_2_, PGI_2_, TXA_2_, and TXB_2_). Moreover, 5-lipoxygenase (5-LOX) metabolizes arachidonic acid to series 4 leukotrienes (LTB_4_, LTC_4_, LTD_4_, and LTE_4_) [18,31]. Prostaglandins, series 2 thromboxanes, and series 4 leukotrienes belong to the pro-inflammatory eicosanoids [31]. However, 15-lipoxygenase (15-LOX) catalyzes the conversion of arachidonic acid to 15-hydroxyeicosatetraenoic acid (15-HETE), whose metabolites are lipoxins. These compounds have anti-inflammatory properties [18,31]. Moreover, 15-HETE can inhibit the formation of 12-HETE, which is a metabolite of 12-LOX’s catalytic action on arachidonic acid [18].

Because DGLA can be metabolically converted via three different enzyme pathways, it is important to determine which enzyme has a higher affinity for arachidonic acid and which metabolites will dominate—pro-inflammatory or anti-inflammatory. Note that γ-linolenic acid, which is one of the main acids contained in evening primrose oil, is an important precursor of DGLA, which is a precursor of anti-inflammatory eicosanoids [32]. It has been shown that GLA or DGLA supplementation causes a modest increase in the prostaglandin E1 (PGE_1_) level in tissues in relation to PGE_2_, but the biological properties of PGE_1_ are about 20 times stronger in comparison to PGE_2_ [24]. However, GLA or DGLA supplementation may cause their conversion to AA and pro-inflammatory eicosanoids. Therefore, it is suggested that the metabolism should be directed to anti-inflammatory eicosanoids. An effective solution is an application of selective Δ-5-desaturase inhibitors, which may stop DGLA’s conversion to AA and its further pro-inflammatory metabolites [24]. Moreover, as a polyunsaturated fatty acid, arachidonic acid undergoes peroxidation to form electrophilic aldehydes with low molecular weight and high reactivity, which may be a cause of the modification of both nucleophilic small molecules and high-molecular weight compounds, such as proteins, lipids, and DNA, which disturbs cellular metabolism [33].

Evening primrose oil has a high content of LA and GLA, and strengthens the epidermal barrier, normalizes the excessive loss of water through the epidermis, regenerates skin, and improves smoothness, after both topical and oral applications [12].

In addition, due to its linoleic acid content, evening primrose oil has a beneficial moisturizing effect on the mucous membrane in acne patients treated with isotretinoin [34]. This implies that skin supplementation with evening primrose oil improves the skin’s water balance, which is weakened by treatment with isotretinoin. Moreover, γ-linolenic acid, contained in large amounts in the oil, is a source of the anti-inflammatory eicosanoids 15-HETrE and PGE_1_, which have anti-proliferative properties that effectively prevent epidermal hyperproliferation [24,34]. In addition, these compounds inhibit the proliferation of smooth muscle cells and prevent the development of atherosclerotic plaque [24].

In recent years, it has been found that γ-linolenic acid is cytotoxic to glioma cells, and it can enhance gamma radiosensitivity [35,36]. It has been suggested that this effect is related to the accumulation of the toxic products of lipid peroxidation, which are cytotoxic to glioma cells. In cancer cells responsible for various types of cancer, the over-expression of the human epidermal growth factor receptor 2 (HER-2/neu) oncogene has been observed. This oncogene causes rapid and uncontrolled cell growth. However, γ-linolenic acid leads to an increase in the levels of polyomavirus enhancer activator 3 (PEA3), a transcriptional repressor of human epidermal growth factor receptor 2 (HER-2/neu) in cells, and a decrease in Her-2/neu promoter activity, thus reducing the likelihood of developing breast cancer [37]. Due to the inhibition of Her-2 expression, GLA administered together with transtuzumab, which as a monoclonal antibody binds to the Her-2 receptor, increases the process of the apoptosis of cancer cells and thus increases the effectiveness of pharmacotherapy with transtuzumab [37]. γ-Linolenic acid also causes an increase in the expression of the nm-23 metastasis-suppressor gene in cancer cells, which favors the inhibition of angiogenesis, cancer cell migration, and consequently, cancer metastasis [38,39]. The formation of these changes is also associated with a reduction in the expression of the vascular endothelial growth factor (VEGF), which plays a significant role in cancer (e.g., in the process of tumor angiogenesis) [40]. The above data suggest that evening primrose oil, as a rich source of gamma linolenic acid, supports anti-cancer therapy.

Moreover, it has been found that the oral supplementation of evening primrose oil (EPO) containing both linoleic acid and γ-linolenic acid reduces the inflammatory reaction and eases eye problems such as burning, dryness, and light sensitivity in people with Sjögren’s syndrome [41]. Moreover, GLA reduces the levels of triacylglycerols and low-density lipoprotein (LDL) cholesterol in plasma [42]. It has been suggested that phytosterols, which are present in large quantities in EPO, also contribute to the above action [43].

## 4. Conclusions

After analyzing the chemical composition of evening primrose (*Oenothera biennis*), especially the oil from its seeds and the biological activity of its components, it can be stated that it is a natural preparation supplementing the deficiency of essential fatty acids in the body. Therefore, it is beneficial in the treatment of chronic inflammation. It supports the metabolism of the body at various levels, especially in situations leading to the development of pathological conditions.

## Figures and Tables

**Table 1:** Fatty acid composition of evening primrose oil (EPO) (*Oenothera biennis* L.) [8].

Compound Name	Contents (%)
linoleic acid	73.88 ± 0.09
γ-linolenic acid	9.24 ± 0.05
oleic acid	6.93 ± 0.02
palmitic acid	6.31 ± 0.14
stearic acid	1.88 ± 0.02
vaccenic acid	0.81 ± 0.03
eicosenoic acid	0.55 ± 0.01
eicosanoic acid	0.31 ± 0.03
behenic acid	0.10 ± 0.01

**Table 2:** Phenolic acid composition (mg/kg) in *Oenothera biennis* L. seed [9].

Acid Name	Included in			
	Free	Esters	Glycosides	Total
*p*-hydroxyphenyl acetic	n/a	1.03 ± 0.18	0.26 ± 0.05	1.29 ± 0.19
*p*-hydroxybenzoic	4.12 ± 0.25	0.38 ± 0.07	0.29 ± 0.10	4.79 ± 0.26
2-hydroxy-4-methoxybenzoic	6.52 ± 0.30	n/a	0.83 ± 0.28	7.35 ± 0.41
caffeic	6.48 ± 0.29	0.80 ± 0.14	n/a	7.51 ± 0.33
hydroxycaffeic	n/a	0.77 ± 0.18	n/a	0.77 ± 0.18
*m*-coumaric	4.90 ± 0.45	0.83 ± 0.21	n/a	5.73 ± 0.50
*p*-coumaric	1.32 ± 0.10	1.96 ± 0.23	0.06 ± 0.06	3.34 ± 0.25
ferulic	4.08 ± 0.30	0.72 ± 0.09	0.22 ± 0.06	5.02 ± 0.32
gallic	1.87 ± 0.22	7.03 ± 0.82	5.91 ± 1.56	14.81 ± 1.78
protocatechuic	50.28 ± 0.77	10.96 ± 0.34	2.16 ± 2.42	63.40 ± 2.56
vanillic	5.22 ± 0.28	0.06 ± 0.02	0.83 ± 0.28	7.35 ± 0.41
veratric	n/a	0.41 ± 0.03	0.47 ± 0.15	0.88 ± 0.15
homoveratric	n/a	0.43 ± 0.06	n/a	0.43 ± 0.06
salicylic	1.15 ± 0.04	1.40 ± 0.18	n/a	2.55 ± 0.18

n/a—not available.

**Table 3:** Sterol content of EPO (*Oenothera biennis* L.) [8].

Compound Name	Contents (mg/kg of Oil)
β-sitosterol	7952.00 ± 342.25
kampesterol	883.32 ± 0.45
Δ_5_-avenasterol	429.65 ± 75.20
sitostanol	167.01 ± 39.77
clerosterol	120.44 ± 0.12
Δ_5_-24-estigmastadienol	94.60 ± 5.68
Δ_7_-estigmasterol	38.17 ± 14.33
Δ_7_-avenasterol	27.80 ± 16.07

**Table 4:** Macroelements and microelements contributing to seed ash [10].

**Macroelements**	**Contents (mg/100g of ash)**
calcium	1800
magnesium	530
potassium	460
sodium	18
phosphorus	410
**Microelements**	**Contents (mg/100g of ash)**
iron	39
zinc	7
copper	1.1
manganese	0.5

**Table 5:** Biological effect and occurrence of selective eicosanoids [18,44,45].

	Metabolite	Biological Activity	Occurrence
**anti-inflammatory**	PGE_1_	- anti-inflammatory - anti-proliferatory	keratinocytes fibroblasts sebocyte
	15-HETrE	- anti-inflammatory - anti-proliferatory	keratinocytes fibroblasts
	13-HODE	- anti-inflammatory - anti-proliferatory	keratinocytes fibroblasts
	15-HETE	- anti-inflammatory (lipoxin precursor) - anti-proliferatory - counteracts 12-HETE and LTB_4_ effects - induces leukocyte chemotaxis	keratinocytes fibroblasts
	LXA_4_ LXB_4_	- anti-inflammatory - LXA_4_ inhibits expression of interleukin 6 (IL-6) and interleukin 8 (IL-8) - LXA_4_ inhibits proliferation	neutrophils
**inflammatory**	PGE_2_	- proliferatory - chemotaxis - immunosuppression	keratinocytes fibroblasts
	5-HETE	- chemotaxis	keratinocytes
	LTB_4_	- chemotaxis	leukocytes keratinocytes in chronic dermatitis (psoriasis, atopic dermatitis)
	Cys-LT (LTC_4_ LTD_4_ LTE_4_)	- leukocyte activators - chemotaxis	leukocytes in chronic dermatitis (psoriasis, atopic dermatitis)
	12-HETE	- proliferatory - chemotaxis	keratinocytes fibroblasts Langerhans cells in chronic dermatitis (psoriasis)

Cys-LT: cysteinyl leukotrienes; LTC_4_: leukotriene C4, LTD_4_: leukotriene D4; LTE_4_—leukotriene E4.
